# IVTA as Adjunctive Treatment to PRP and MPC for PDR and Macular Edema: A Meta-Analysis

**DOI:** 10.1371/journal.pone.0044683

**Published:** 2012-09-04

**Authors:** Lei Liu, Xiaomei Wu, Jin Geng, Zhe Yuan, Lei Chen

**Affiliations:** 1 Department of Ophthalmology, The First Hospital of China Medical University, Shenyang, People's Republic of China; 2 Department of Clinical Epidemiology and Evidence Medicine, The First Hospital of China Medical University, Shenyang, People's Republic of China; 3 The Key Laboratory of Endocrine Diseases in Liaoning Province, The First Hospital of China Medical University, Shenyang, People's Republic of China; 4 Liaoning Diabetic Eye Center, The First Hospital of China Medical University, Shenyang, People's Republic of China; University of Texas Health Science Center at San Antonio, United States of America

## Abstract

**Background:**

To quantify the effect of a combination treatment of intravitreal triamcinolone acetonide (IVTA) injection, panretinal photocoagulation (PRP), and macular photocoagulation (MPC) in patients with proliferative diabetic retinopathy (PDR) and diabetic macular edema (DME).

**Methodology/Principal Findings:**

We conducted a meta-analysis and searched for reports concerning IVTA injection combined with PRP for the treatment of PDR and DME using Medline, EMbase, Web of Science, the Cochrane Library, and Google according to Cochrane evaluation guidelines. The quality of the reports was evaluated using the Jadad score. Only four studies were ultimately included in this meta-analysis and the fixed-effects model was used. Treatment with IVTA injection combined with PRP and MPC significantly improved BCVA (*p*<0.001) from one to six months, compared with PRP and MPC alone. There was a statistically significant mean difference in central macular thickness (CMT), at the one-month follow-up (*p*<0.001). No evidence of publication bias was present. There was a low level of heterogeneity in this group of studies.

**Conclusions/Significance:**

This meta-analysis indicates that IVTA injection combined with PRP and MPC results in an improvement of BCVA, and CMT reduction in patients with PDR and DME.

## Introduction

Diabetic retinopathy (DR) is one of the foremost causes of blindness in the working age group [1]. Many patients with proliferative diabetic retinopathy (PDR) may also have diabetic macular edema (DME) due to DR in either eye [2]. As is well known, panretinal photocoagulation (PRP) can reduce the risk of severe visual loss in patients with high-risk PDR [3], [4], [5], but PRP sometimes causes or aggravates macular edema (ME), which is the main cause of visual reduction. Even though macular photocoagulation (MPC) (focal/grid laser photocoagulation) seems to effectively treat ME, the timing of PRP may be delayed because it should be performed after sufficient stabilization of ME [6].

A previous study demonstrated that IVTA injection may improve inflammatory, edematous, and neovascular ocular conditions [7], and IVTA injection has been used to treat ME combined with MPC. A hypothesis was formed that IVTA injection may have an additive effect with the standard treatment (PRP and MPC) with respect to visual acuity (VA) improvement, and central macular thickness (CMT) reduction [8]. Studies were carried out to clarify this, but the conclusions were inconsistent.

To quantify the effect of a combination treatment of IVTA injection and PRP and MPC on patients with PDR and DME, we performed a meta-analysis of randomized controlled trials (RCTs), and assessed the BCVA and CMT after intravitreal triamcinolone combined with PRP and MPC laser-refractory PDR and DME.

## Methods

This meta-analysis was performed according to a predetermined protocol described previously in QUOROM statement and MOOSE recommendations [9], [10].

### Search Strategy

Two researchers (Lei Liu, Jin Geng) independently searched the literature using the following electronic databases: MEDLINE, EMBASE, Web of Science, Google (Scholar), and the Cochrane Library. Manual searching of bibliographies was also performed. All published and internet-accessible articles (as on the 10 April 2012) were considered. For maximum sensitivity, the search strategy for free text and thesaurus terms included ‘triamcinolone’, ‘diabetic retinopathy’, and ‘panretinal photocoagulation’. The dates for the MEDLINE search were 1950 to April 2012, the dates for the EMBASE search were 1998 to April 2012, and the Cochrane Library and Google (Scholar) were accessed on 10 April 2012. Full articles were retrieved, when titles and/or abstracts met the study objective. All published studies that evaluated IVTA injection combined with PRP and MPC treatment for PDR were included, if they met the criteria. The search included only RCTs. At least one or more clinical outcome representing intraoperative and/or postoperative outcome parameters had to have been assessed and published. There was no language restriction on the publications.

### Inclusion and Exclusion Criteria

Articles potentially eligible for inclusion in this meta-analysis were RCTs on IVTA injection combined with PRP and MPC therapy for PDR and DME published up to April 2012.

Exclusion criteria were: causes of macular edema other than diabetic retinopathy, when triamcinolone was not used as an adjunct to PRP, and republication articles. In addition, articles were excluded if they did not satisfy one or more inclusion criteria.

### Data Extraction

Data were extracted from each article using a standardized form including: (1) general data: title, authors, publication date, resource; (2) subjects, intervention measures, quality control; and (3) outcomes.

The four trials that were included in the meta-analysis presented comparable data suitable for quantitative statistical analysis.

### Outcome measures

BCVA of logarithm of the minimum angle of resolution (logMAR) units, CMT, intraocular presser (IOP), standard central macular thickness (SCMT), total macular volume (TMV), any changes in VA compared with the pre-injection level, and complications after therapy, were evaluated following IVTA injection combined with PRP and MPC for treatment of PDR and DME in the various outcomes from the different articles.

### Quality Assessment of Retrieved Articles

Methodological quality was assessed by methods of random allocation, allocation sequence concealment, blinding and missing data on follow-up using the Jadad Score [11]. High quality was previously defined as a Jadad score more than three.

### Data Analysis

RevMan 5.0 statistical software offered by the Cochrane collaboration net was used to analyze the data. The treatment effects were calculated as weighted averages of the mean difference in logMAR visual acuity between study and control groups (weighted mean difference method).

All meta-analyses were evaluated for heterogeneity using the Q statistic of the Chi-square test (χ^2^) value test and *I* ^2^ test [12].

*I^2^* estimated the percentage of the total variance in all of the data under consideration that was related to heterogeneity. The authors suggested using 25%, 50%, and 75% to indicate low, moderate or high levels of heterogeneity. If there was a moderate or high level of heterogeneity, a random-effects meta-analysis was performed by the DerSimonian and Laird method unless using the fixed-effects models. Publication bias was assessed by visually inspecting a funnel plot. A *p* value less than 0.05 was considered statistically significant [13], [14].

## Results

**Figure 1:**
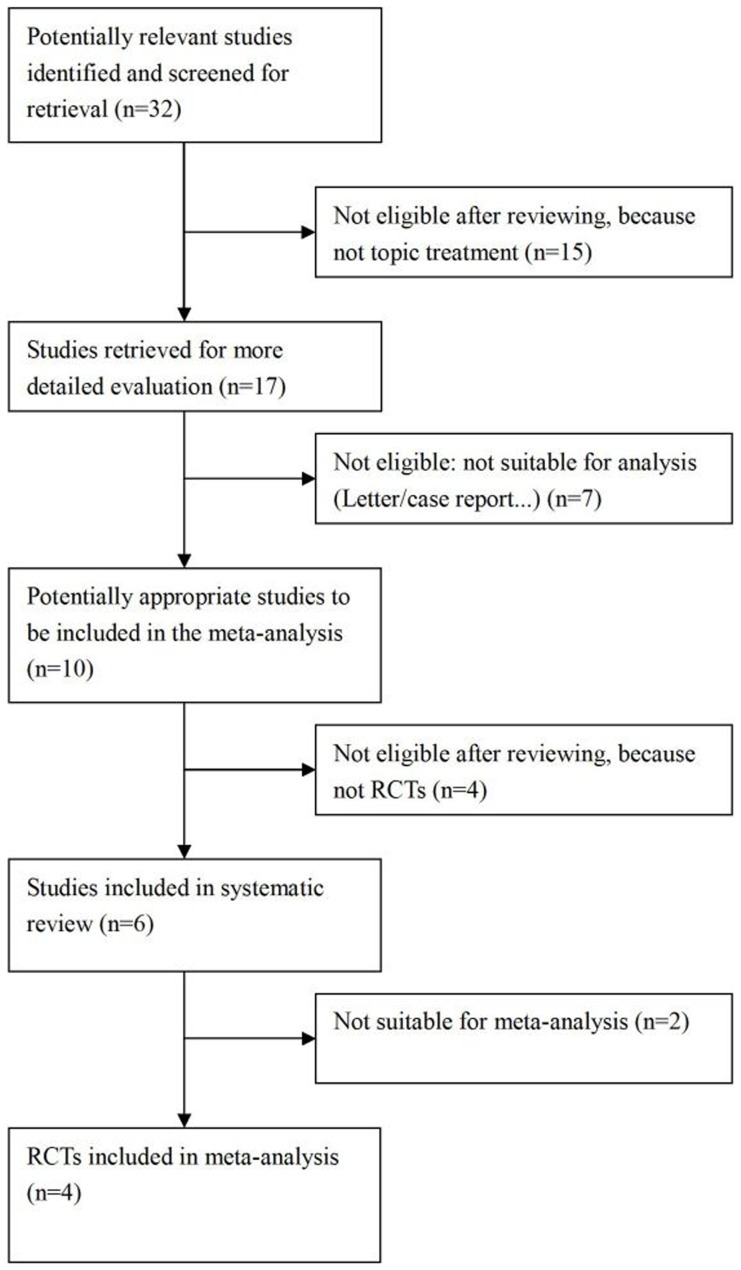
Flow chart demonstrating the process for study inclusion in the meta-analysis.

A total of 32 articles that were potentially relevant were identified and screened for retrieval (Table 1). After systematic review, only four studies were ultimately included in the meta-analysis. The progress for study inclusion is shown in Figure 1. One of the trials was performed in Europe, two in Asia, and the other was in South America. Two studies were unclear blinding, and 59 eyes were included in the meta-analysis. The dose for the IVTA injection was 4 mg. Mean follow-up was 8.25 (SD 4.5) months. BCVA and CMT were common outcomes, and we evaluated these in our meta-analysis. The characteristics for each study are shown in Table 2.

**Table 1:** A list of potentially relevant reports and the reasons for exclusion.

No.	Title	Author	Journal	Publication year; Volume∶Page.	Reasons for exclusion/inclusion.
1	Intravitreal triamcinolone acetonide as an adjuvant therapy to panretinal photocoagulation for proliferative retinopathy with high risk characteristics in type 1 diabetes: case report with 22 weeks follow-up	Kytö JP et al.	Acta Ophthalmol Scand.	2005;83∶605–608.	Case-report
2	Panretinal photocoagulation and intravitreal triamcinolone acetonide injection in four diabetic patients.	Er H.	Retina.	2005;25∶675.	Case-report
3	Combined intravitreal injection of triamcinolone acetonide and panretinal photocoagulation for concomitant diabetic macular edema and proliferative diabetic retinopathy.	Zacks DN et al.	Retina.	2005;25∶135–140.	Case-report
4	Intravitreal triamcinolone as an adjunct to standard laser therapy in coexisting high-risk proliferative diabetic retinopathy and clinically significant macular edema.	Mirshahi A et al.	Retina.	2010;30∶254–259.	Inclusion
5	Combined laser and intravitreal triamcinolone for proliferative diabetic retinopathy and macular edema: one-year results of a randomized clinical trial.	Maia OO Jr et al.	Am J Ophthalmol.	2009;147∶291–297.e2.	Inclusion
6	Laser photocoagulation combined with intravitreal triamcinolone acetonide injection in proliferative diabetic retinopathy with macular edema.	Choi KS et al.	Korean J Ophthalmol.	2007;21∶11–17.	Inclusion
7	Triamcinolone as adjunctive treatment to laser panretinal photocoagulation for proliferative diabetic retinopathy.	Bandello F et al.	Arch Ophthalmol.	2006;124∶643–650.	Inclusion
8	Combined laser and intravitreal triamcinolone for proliferative diabetic retinopathyand macular edema.	Kumar V et al.	Am J Ophthalmol.	2009;148∶171.	Letter
9	Randomised controlled trial of posterior sub-Tenon triamcinolone as adjunct to panretinal photocoagulation for treatment of diabetic retinopathy.	Unoki N et al.	Br J Ophthalmol.	2009;93∶765–770.	Not topic treatment
10	Posterior sub-Tenon's capsule injection of triamcinolone acetonide prevents panretinal photocoagulation -induced visual dysfunction in patients with severe diabetic retinopathy and good vision.	Shimura M et al.	Ophthalmology.	2006;113∶381–387.	Not topic treatment
11	Intravitreal triamcinolone as adjunctive treatment to laser panretinal photocoagulation for concomitant proliferative diabetic retinopathy and clinically significant macular oedema.	Margolis R et al.	Acta Ophthalmol.	2008;86∶105–110.	Not RCT
12	Intravitreal triamcinolone as an adjunct in the treatment of concomitant proliferative diabetic retinopathy and diffuse diabetic macular oedema. Combined IVTA and laser treatment for PDR with CSMO.	Kaderli B et al.	Int Ophthalmol.	2005;26∶207–214.	Not RCT
13	Periocular abscess caused by Pseudallescheria boydii after a posterior subtenon injection of triamcinolone acetonide.	Oh IK et al.	Graefes Arch Clin Exp Ophthalmol.	2007;245∶164–166.	Not RCT
14	Complete resorption of retinal hemorrhages in idiopathic thrombocytopenic purpura.	Meyer CH et al.	Eur J Ophthalmol.	2007;17∶128–129.	Not suitable for meta-analysis
15	Two-year results of a randomized trial of intravitreal bevacizumab alone or combined with triamcinolone versus laser in diabetic macular edema.	Soheilian M et al.	Retina.	2012;32∶314–321.	Not suitable for meta-analysis
16	Current trends in the pharmacotherapy of diabetic retinopathy.	Kumar B et al.	J Postgrad Med	2012;58∶132–139.	Review
17	Corticosteroid use for diabetic macular edema: old fad or new trend?	Stewart MW	Curr Diab Rep	2012;12∶364–375.	Review
18	Randomized trial evaluating short-term effects of intravitreal ranibizumab or triamcinolone acetonide on macular edema after focal/grid laser for diabetic macular edema in eyes also receiving panretinal photocoagulation.	Diabetic Retinopathy Clinical Research Network et al.	Retina	2011;31∶1009–1027.	Not topic treatment
19	Factors associated with improvement and worsening of visual acuity 2 years after focal/grid photocoagulation for diabetic macular edema.	Aiello LP et al.	Ophthalmology	2010;117∶946–953.	Not topic treatment
20	Intravitreal triamcinolone and bevacizumab as adjunctive treatments to panretinal photocoagulation in diabetic retinopathy.	Cho WB et al.	Br J Ophthalmol.	2010;94∶858–863.	Not topic treatment
21	Exploratory analysis of diabetic retinopathy progression through 3 years in a randomized clinical trial that compares intravitreal triamcinolone acetonide with focal/grid photocoagulation.	Bressler NM et al.	Arch Ophthalmol.	2009;127∶1566–1571.	Not topic treatment
22	Intravitreal bevacizumab (Avastin) therapy for persistent diffuse diabetic macular edema.	Haritoglou C et al.	Retina.	2006;26∶999–1005.	Not topic treatment
23	Opaque coating of an intraocular lens and regression of iris neovascularization following injection of triamcinolone acetonide into the anterior chamber.	Chen SD et al.	Clin Experiment Ophthalmol.	2006;34∶803–805.	Not topic treatment
24	Intravitreal triamcinolone for macular detachment following panretinal photocoagulation.	Gharbiya M et al.	Eye (Lond).	2005;19∶818–820.	Not topic treatment
25	Intravitreal triamcinolone acetonide for florid proliferative diabetic retinopathy.	Bandello F et al.	Graefes Arch Clin Exp Ophthalmol.	2004;242∶1024–1027.	Not topic treatment
26	Efficacy of intravitreal triamcinolone after or concomitant with laser photocoagulation in nonproliferative diabetic retinopathy with macular edema.	Aydin E et al.	Eur J Ophthalmol.	2009;19∶630–637.	Not topic treatment
27	Cost-Effectiveness Analysis of Ranibizumab Plus Prompt or Deferred Laser or Triamcinolone Plus Prompt Laser for Diabetic Macular Edema.	Dewan V et al.	Ophthalmology.	2012;[Epub ahead of print]	Not topic treatment
28	Combination of vitrectomy, IVTA, and laser photocoagulation for diabetic macular edema unresponsive to prior treatments; 3-year results.	Kim YT et al.	Graefes Arch Clin Exp Ophthalmol.	2012;250∶679–684.	Not topic treatment
29	Nine-month results of intravitreal bevacizumab versus triamcinolone for the treatment of diffuse diabetic macular oedema: a retrospective analysis.	Kook PE et al.	Acta Ophthalmol.	2011;89∶769–773.	Not topic treatment
30	Intravitreal triamcinolone versus laser photocoagulation as a primary treatment for diabetic macular oedema – a comparative pilot study.	Norlaili M et al.	BMC Ophthalmol.	2011;11∶36.	Not topic treatment
31	Cataract surgery and diabetes.	Shah AS et al.	Curr Opin Ophthalmol.	2010;21∶4–9.	Review
32	Panretinal photocoagulation and intravitreal triamcinolone acetonide for the management of proliferative diabetic retinopathy with macular edema.	Zein WM et al.	Retina.	2006;26∶137–142.	Not RCT

IVTA =  intravitreal injection of triamcinolone acetonide; RCT =  randomized clinical trial; PDR =  proliferative diabetic retinopathy; CSMO =  clinically significant macular oedema.

**Table 2:** Characteristics of randomized controlled trials (RCTs) evaluating IVTA combined with PRP and MPC for treating PDR and DME.

	Country	Published Year	Exposure for study group	Exposure for control group	Dose	n (eyes)	Mean age ± SD (years)	Gender	Type of Diabetes	Blinding of intervention	Time of follow up	Outcomes	Effective	Quality assessment (Jadad score)
Bandello F et al.^15^	Italy	2006	IVTA + PRP + MPC	PRP + MPC	4 mg	9	47.5±2.3	8 men,1 woman	3 type 1, 6 type2	yes	10 to 15 days after injection and at 3,6,9,12 months	BCVA + FA + CMT	yes	4
Choi KS et al.^8^	Korea	2007	IVTA + PRP + MPC	PRP + MPC	4 mg	10	NA	NA	NA	yes	2 weeks, 1,2,3 months	BCVA + FA + CMT	yes	3
Maia OO Jr et al.^16^	Brazil	2009	PRP + MPC + IVTA	PRP + MPC	4 mg	22	61.9±5.3	10 men,12 woman	type 2	yes	1,3,6,9,12 months	BCVA + CMT + TMV	yes	4
Mirshahi A et al.^17^	Iran	2010	IVTA + PRP + MPC	PRP+ MPC	4 mg	18	55.6±6.5	NA	type 2	yes	1,4,and 6 months	BCVA + IOP + CMT + SMCT + VA*	no	5

IVTA =  intravitreal injection of triamcinolone acetonide; PRP =  panretinal photocoagulation; BCVA =  best corrected visual acuity; FA =  fluorescein angiography; CMT =  central macular thickness; NA =  not available; RAFL =  perimetric area of fluorescein leakage; SMCT =  standardized changes in macular thickness; MPC =  macular photocoagulation; TMV =  total macular volume; IOP =  intraocular pressure.

* any change in VA compared with the pre-injection level.

**Figure 2:**
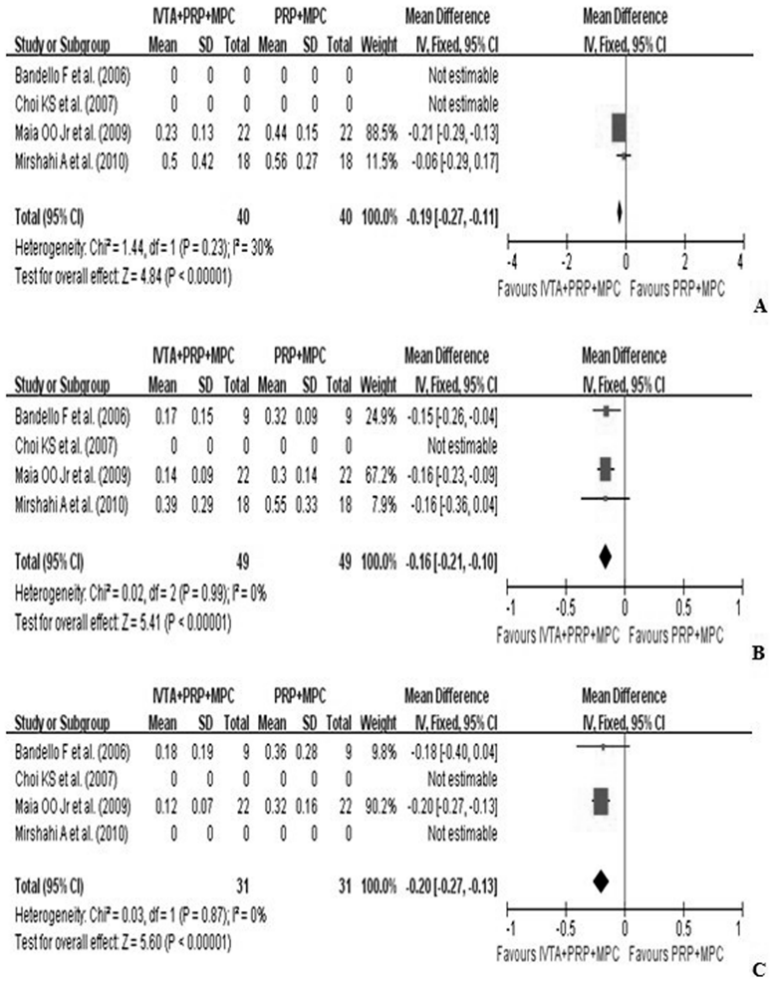
Forest plot displaying the pooled summary estimates of BCVA in the study group versus the control group: A at 1 month, B at 6 months, and C at 12 months. BCVA =  best-corrected visual acuity; IVTA =  intravitreal injection of triamcinolone acetonide; MPC =  macular photocoagulation; PRP =  pan-retinal photocoagulation; SD =  standard deviation; IV =  weighted mean difference; CI =  confidence interval; df =  degrees of freedom; Chi^2^ =  chi-square statistic; p =  p value; I^2^ =  I-square heterogeneity statistic; Z =  Z statistic.

VA was the most important criterion for evaluating efficacy. In all of the studies, BCVA was reported as a mean change in logMAR units, and measured by logMAR scale from baseline to follow-up months. Pooling the results revealed that at one-month follow-up, treatment with IVTA injection combined with PRP and MPC (study group) significantly improved BCVA compared with PRP and MPC alone (control group) (WMD, −0.19; 95%CI, −0.27 to −0.11; p<0.01). Similar efficacy was found at six months (WMD, −0.16; 95%CI, −0.21 to −0.10; p<0.01), and 12 months (WMD, −0.20; 95%CI, −0.27 to −0.13; p<0.01) (Figure 2).

**Figure 3:**
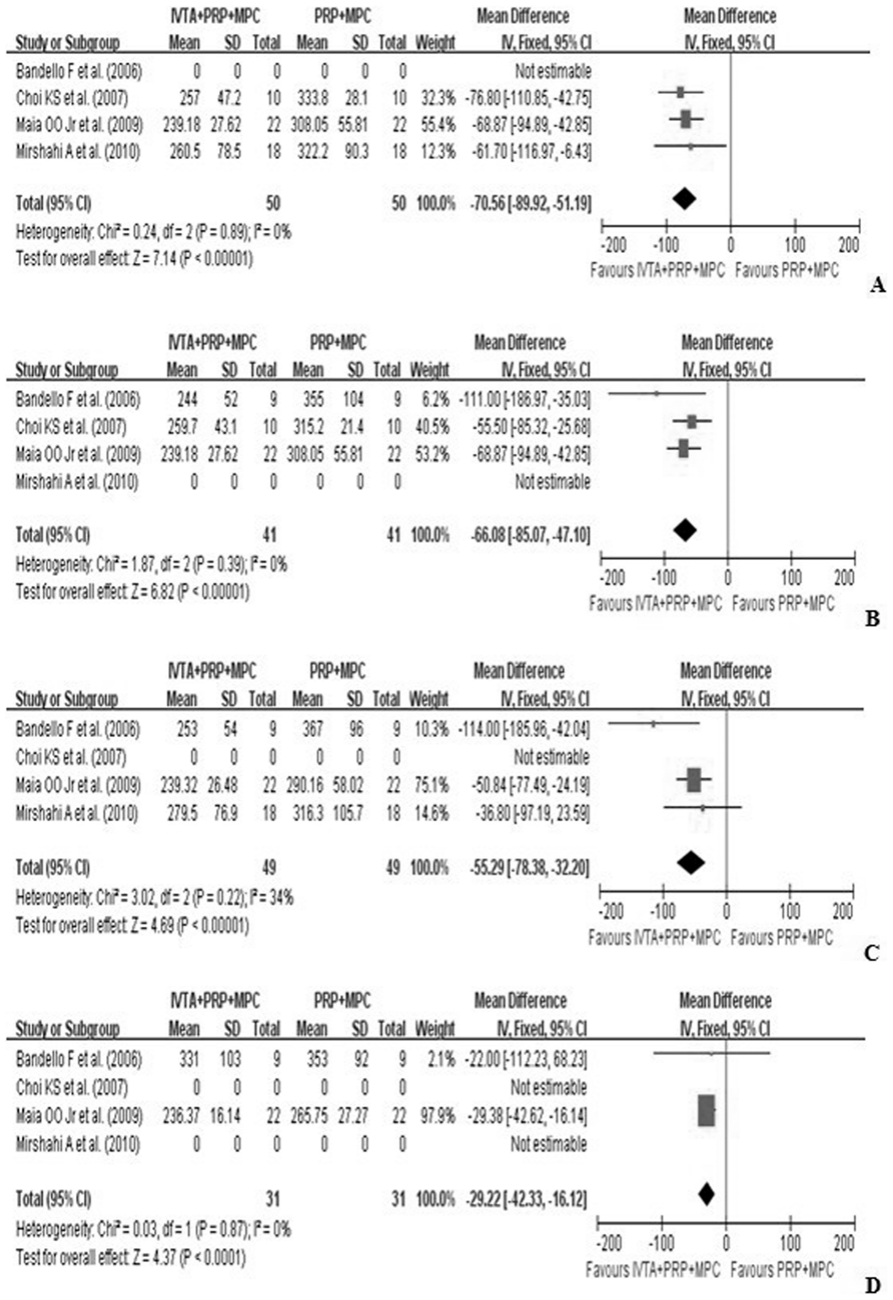
Forest plot displaying the pooled summary estimates of CMT in the study group versus the control group: A at 1 month, B at 3 months, C at 6 months, and D at 12 months. CMT =  central macular thickness; IVTA =  intravitreal injection of triamcinolone acetonide; MPC =  macular photocoagulation; PRP =  pan-retinal photocoagulation; SD = standard deviation; IV =  weighted mean difference; CI =  confidence interval; df =  degrees of freedom; Chi^2^ =  chi-square statistic; p =  p value; I^2^ =  I-square heterogeneity statistic; Z =  Z statistic.

CMT is considered a strong prognostic measure for levels of ME, so it was also assessed in this meta-analysis. CMT was reported as the mean change from baseline to follow-up months and was measured by optical coherence tomography (OCT). The pooled results revealed that at one-month follow-up, treatment in the study group significantly improved CMT, compared with the control group (WMD, −70.56; 95%CI, −89.92 to −51.19; p<0.01). Similar efficacy was found at three months (WMD, −66.08; 95%CI, −85.07 to −47.10; p<0.01) and six months follow-up (WMD, −55.29; 95%CI, −78.38 to −32.20; p<0.01) (Figure 3).

**Figure 4:**
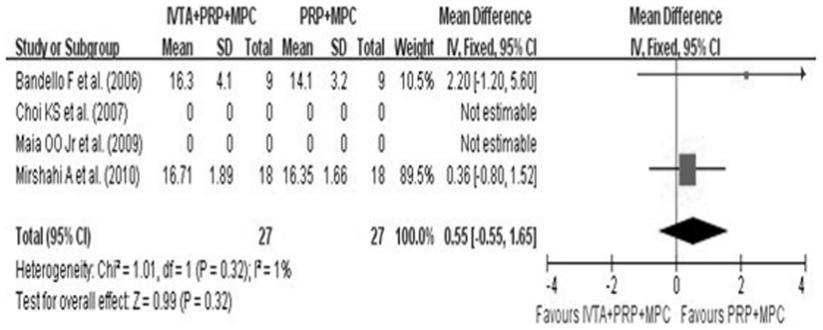
Forest plot displaying the pooled summary estimates of IOP in the study group versus control group at 6 months. IOP =  intraocular pressure; IVTA =  intravitreal injection of triamcinolone acetonide; MPC =  macular photocoagulation; PRP =  pan-retinal photocoagulation; SD =  standard deviation; IV =  weighted mean difference; CI =  confidence interval; df =  degrees of freedom; Chi^2^ =  chi-square statistic; p =  p value; I^2^ =  I-square heterogeneity statistic; Z =  Z statistic.

There were no complications in these four studies. IOP was mentioned in all four studies, thus IOP was assessed in the meta-analysis. However, at six months follow-up, there were no significant differences in IOP in the study group compared with the control group (WMD, 0.55; 95%CI, −0.55.to 1.65; p<0.01) (Figure 4). In addition, only one study reported that two ITVA eyes had significant cataract progression.

**Figure 5:**
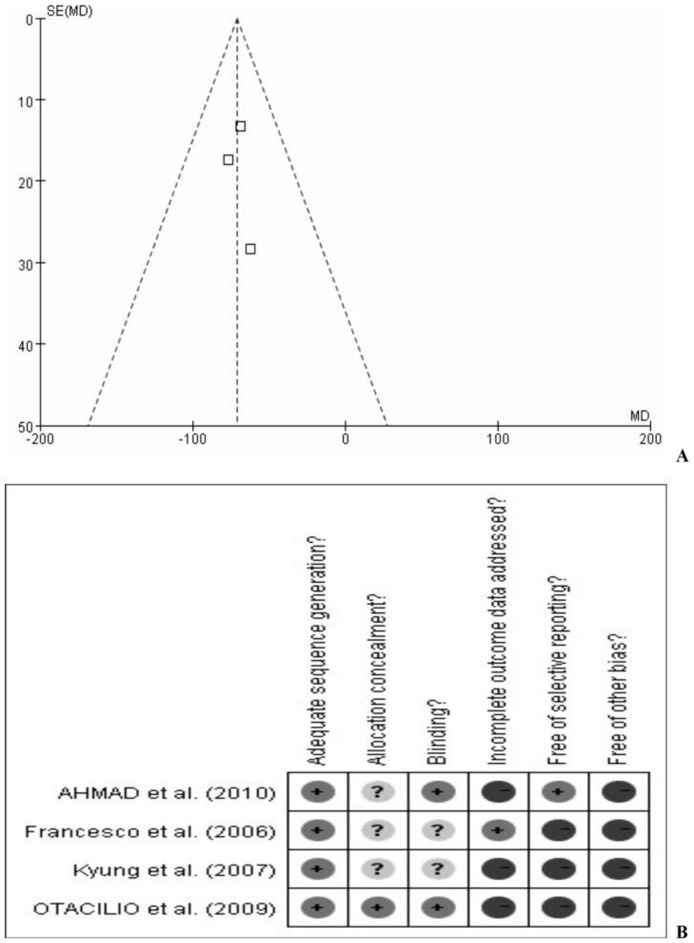
Funnel plot of randomized controlled trials(A). Risk of bias summary across all studies; ‘+’: present; ‘−’: absent; ‘?’: questionable(B).

All study comparisons passed the test of heterogeneity, as previously defined. Fixed-effects models were used for the meta-analysis. There was no significant publication bias in this meta-analysis. The funnel plot of randomized controlled trials and a risk of bias for publication summary following a bias-risk evaluation are shown in Figure 5.

## Discussion

This meta-analysis revealed that ITVA combined with PRP and MPC was an effective treatment for PDR and DME. Three of the reported studies (Bandello F et al. [15], Choi KS et al. [8], and Maia OO Jr et al. [16]) confirmed that ITVA combined with PRP and MPC was effective for treating PDR and DME, but the conclusions of Mirshahi A et al. [17] were not in accordance. Thus, we conducted this meta-analysis to clarify whether ITVA injection combined with PRP and MPC was effective for the treatment of PDR and DME.

TA is commonly used to treat ME in the clinical situation, and IVTA has been shown to be useful in the reduction of CMT caused by DME [18].

As we know, new vessel formation stimulated by vascular endothelial growth factor (VEGF) is the major pathology underlying PDR. In addition, studies have suggested that IVTA is clinically effective in inhibiting the metabolic pathway of VEGF, and in anti-inflammatory, edematous, neovascular, and proliferative disorders [19], [20]. PRP is recommended as an effective method to prevent neovascularization and progression of PDR, but aggravation of ME with a decrease in VA can occur in DR after PRP. There has thus been an initiative to study the effect of IVTA combined with PRP and MPC on PDR and DME in patients.

As a result of structural changes caused by poorly controlled diabetes, Mirshahi A et al. reported that IVTA combined with PRP and MPC did not have a significant beneficial effect on BCVA improvement and CMT reduction in coexisting PDR and DME, compared with PRP and MPC as a standard treatment in these patients [17].

Our meta-analysis for all logMAR scales of BCVA during follow-up months revealed that IVTA injection combined with PRP and MPC could improve BCVA for PDR and DME. CMT is an important criterion for evaluating ME and was assessed during follow-ups. We revealed that IVTA injection combined with PRP and MPC could reduce CMT in PDR and DME. The reason for the efficacy of TA is that it improves inflammation and changes in retinal blood flow associated with photo-oxidative reactions induced by laser-tissue interactions [21]. Wilson et al. reported that an intravitreal steroid in an animal (rabbit) model reduced the blood retinal barrier breakdown that was induced by retinal photocoagulation [22].

In the study by Maia OO Jr et al. [16] IVTA was carried out before PRP and MPC. However, in the other three studies IVTA was carried out after PRP and MPC. Therefore, the study by Maia OO Jr et al. may have introduced methodological bias in the analysis, but after considering all the other inclusion criteria, we included it in this meta-analysis. Prospective studies are needed to evaluate the efficacy of different methods.

There were some limitations to this study: (1) Only a small number of trials were enrolled in this meta-analysis; (2) There were short, and different follow-up times for observation; (3) Only two common outcomes were evaluated; (4) As we cannot attempt to gain access to unpublished results, publication bias cannot be fully excluded.

As far as we know, this is the first systematic review specifically answering the question of whether the combined treatment method of IVTA injection with PRP and MPC is effective for treating PDR and DME. Even with the limitations, we feel the conclusions of this meta-analysis are clinically useful for treatment considerations.

In conclusion, IVTA injection combined with PRP and MPC may represent a new strategy to more effectively treat PDR and DME. RCTs with larger sample sizes, possibly multicenter trials, and longer follow-up times are needed to better evaluate the benefits and safety of IVTA injection combined with PRP and MPC for PDR and DME.
